# Targeting immune-checkpoint inhibitor resistance mechanisms by MEK inhibitor and agonist anti-CD40 antibody combination therapy

**DOI:** 10.15698/cst2020.10.233

**Published:** 2020-08-06

**Authors:** Daniel Baumann, Rienk Offringa

**Affiliations:** 1 Department of Molecular Oncology of Gastrointestinal Tumors, German Cancer Research Center Heidelberg, Heidelberg, Baden-Wuerttemberg, 69120, Germany.; 2 Department of Surgery, Heidelberg University Hospital, Heidelberg, Baden-Wuerttemberg, 69120, Germany.

**Keywords:** cancer immunotherapy, immune checkpoint blockade, combinatorial immunotherapy, immunogenic cell death, cytostatic agents, MEK inhibition, MAPK, CD40, agonist

## Abstract

The widespread application of immune-checkpoint blockade (ICB) has resulted in unprecedented response rates in patients with immunogenic cancers, such as melanoma and lung cancer. However, sub-groups of patients with these indications do not respond to ICB, and the same applies to patients with other cancer types. Mechanisms of resistance to ICB include low tumor immunogenicity associated with low T cell infiltration (‘cold' tumors), suppression of anti-tumor immunity by immunosuppressive cells in the tumor microenvironment (TME), lack of antigen-presentation and immune escape (e.g. by downregulation of MHC-I on tumor cells) as well as oncologic pathways that suppress immune responses. Combination strategies, involving cytostatic drugs, harbor the potential to overcome refractoriness to immunotherapy. However, suppression of immune cell function by cytostatic drugs may limit the efficacy. In our study, we show that combination treatment of targeted inhibition of mitogen-activated protein kinase (MAPK) kinase (MEK) and agonist immunostimulatory anti-CD40 antibody (Ab) is particularly suitable in counteracting aforementioned ICB resistance mechanisms (Fig. 1).

## IDENTIFYING SUITABLE CYTOSTATIC DRUGS FOR COMBINATORIAL IMMUNOTHERAPY

The main dilemma in finding combinations of cytostatic and immunostimulatory drugs that act synergistically is as follows. On the one hand, the aim is to induce tumor cell death, not merely stasis, thereby feeding the immune system with tumor antigen. On the other hand, it is essential not to paralyze the function of tumor antigen cross-presenting DCs and of the anti-tumor T cell response. We therefore tested multiple cytostatic drugs for their impact on tumor cell killing as well as in *in vivo* immunization model in which T cell priming requires antigen cross-presentation by activated DCs, using agonist anti-CD40 Abs as the dendritic cell (DC)-activating signal. These assays revealed two things. First, MEK inhibitors (MEKi) are particularly suitable for targeting Kras-driven pancreatic ductal adenocarcinoma (PDA) tumor cells as well as other tumor cells lines with activated MAPK/ERK signaling. Secondly, gemcitabine (GEM) and temozolomide, chemotherapeutics drugs generally considered mild and therefore applied in the context of immunostimulatory antibodies in pre-clinical and clinical settings, strongly suppressed DC-dependent T cell priming and expansion. In contrast, a variety of small molecule drugs targeting mediators of oncogenic signaling, in particular MEK, PI3K and mutant BRAF, showed no or only minor suppressive impact in this setting, suggesting that these drugs may be a better match with immune-oncology drugs. Notably, MEKi did significantly inhibit antigen-specific T cell activation in our *in vitro* assays, in line with the general notion that MAPK/ERK is involved in T cell activation. This implies that in the *in vivo* immunization setting signals downstream of CD40-ligation can overcome this inhibitory effect on T cells, possibly through T cell costimulatory signals provided by activated DCs (**Fig. 2A**).

**Figure 1:**
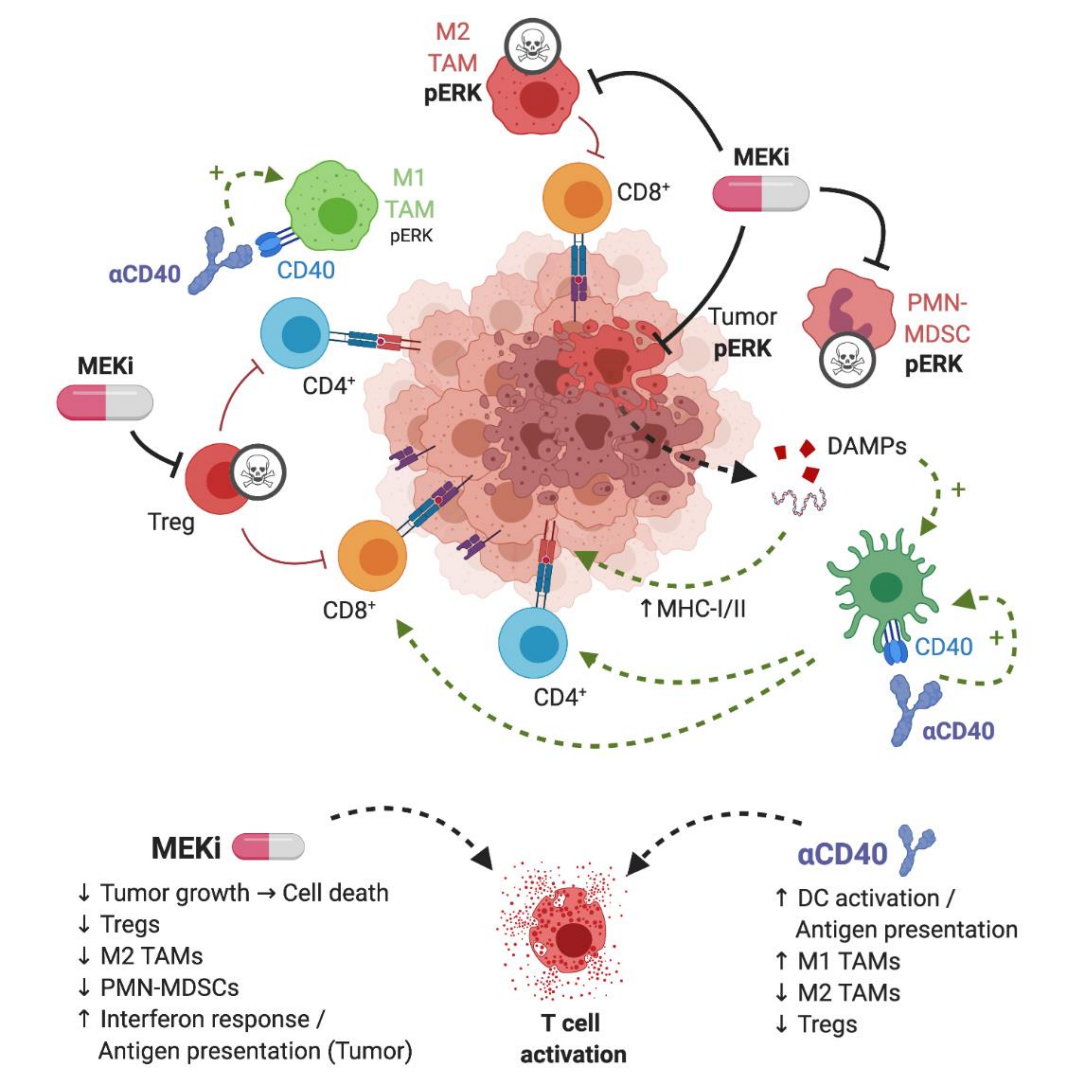
FIGURE 1: Graphical representation of MEKi/CD40 Ab antitumor mechanism. MEKi can play a dual role in anti-tumor immune responses by inducing immunogenic cell death of tumor cells and by eliminating immunosuppressive immune cells in the TME, in particular T-regulatory cells, M2-type macrophages and MDSCs. Agonist anti-CD40 provides co-stimulatory signal, increases antigen presentation, and stimulates CD4^+^ and CD8^+^ T cells. These complementary drug actions exert synergistic T cell-dependent anti-tumor effects. Figure created with BioRender.com.

**Figure 2:**
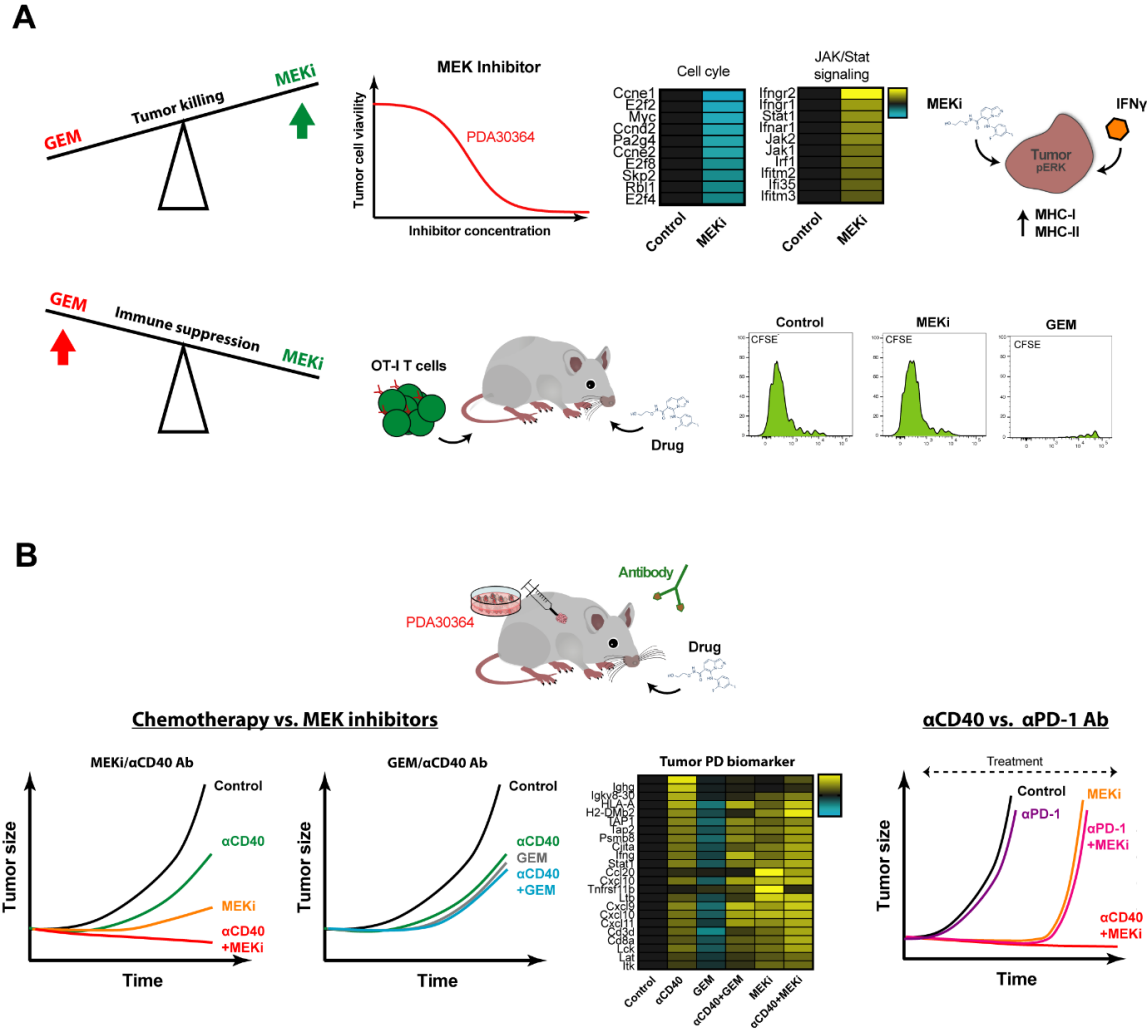
FIGURE 2: Effect of cytostatic drugs on tumor and immune cells and during anti-tumor immunity. **(A)** Effect of MEKi on tumor cell viability, expression of pro-inflammatory genes and MHC-I/-II protein levels in presence of low doses IFNγ. Effect of MEKi and GEM on T cell expansion in *in vivo* OT-I T cell proliferation assays. **(B)** Anti-tumor efficacy and PD biomarker gene signatures of combinations therapies involving cytostatic agents GEM and MEKi and immunostimulatory antibodies targeting CD40 and PD-1.

Another desirable feature of small molecule drugs is that in addition to their anti-proliferative effects, they harbor the capacity to sensitize tumor cells for killing by the immune system. In our experiments with MEKi, we documented a pro-inflammatory gene signature reflected by induction of multiple signaling pathways associated with interferon signaling. Interestingly, when we combined MEKi treatment with low levels of interferon-gamma (IFNγ), as is expected to be released by T cells in the TME upon stimulation, an induction of both MHC class I and MHC class II was observed (**Fig. 2A**). Induction of MHC-II by MEKi treatment is of particular interest for the clinic, with regard to immune escape by downregulation/loss of MHC class I-restricted antigen presentation, as commonly found in various human and experimental tumors, including PDA.

## COMBINATORIAL IMMUNOTHERAPY WITH MEK INHIBITOR AND AGONIST ANTI-CD40 ANTIBODY

In view of the outcome of the *in vivo* immunization assays and our interest in identifying combinatorial regimens for pancreatic cancer, we proceeded by testing the combination of MEKi and agonist anti-CD40 Abs in three different syngeneic tumor models, including a newly developed K-ras driven model for PDA. We observed a strong synergistic anti-tumor effect of MEKi/CD40 Ab, which was accompanied by prominent changes in immune-cell infiltrate in the TME. In line with the proven T cell dependent anti-tumor effect, we observed a dramatic increase in the CD8/Treg ratio by the MEKi/CD40 Ab combination. Interestingly, the single drug groups had complementary effects: Anti-CD40 Ab was the primary driver of CD8^+^ T cell infiltration, whereas both anti-CD40 Ab and MEKi exert suppressive function on Tregs. In addition, MEKi elicited a dramatic reduction in immunosuppressive myeloid cells in the TME, namely CD206^+^ M2-polarized macrophages as well as MDSCs (**Fig. 2B**).

We benchmarked this regimen against the combination of gemcitabine and anti-CD40 Ab that has been explored extensively in this indication, both in the pre-clinical and clinical setting. The outcome of these experiments was in line with the immunization assays, in that the MEKi/CD40 Ab combination was clearly superior to each of the single agent treatments, whereas we did not observe a beneficial effect of combining gemcitabine with anti-CD40 Ab. The picture obtained by extensive pharmacodynamic (PD) biomarker analysis by means of flow cytometry and whole tumor transcriptome analysis closely reflected these findings. In brief, MEKi and CD40 Ab had clear cut synergistic actions in increasing the CD8^+^/regulatory T cell and M1/M2 macrophage ratios, suppressing the tumor growth-related gene signature and enhancing the pro-immunogenic signature, while such indications for drug synergy were lacking in the GEM/CD40 Ab combination. These differences between the MEKi/CD40 Ab combination and the GEM/CD40 Ab benchmark can at least in part be explained by our finding that CD40 Ab can overcome the suppressive impact of MEKi on *in vivo* T cell stimulation in the immunization model, but not that of GEM. However, our whole tumor gene signatures furthermore pointed at an important role of MEKi-induced tumor cell death in this respect, in particular in the mutant K-ras driven PDA model, in that MEKi single agent treatment already induced a pro-inflammatory gene signature associated with IFN pathway activation. As such, the outcome of this combination regimen closely aligns with our intention to induce immunogenic tumor cell death with the cytostatic drug while reprogramming the immune cell profile using the immunostimulatory antibody.

## BENCHMARKING MFKi/CD40 AB TO IMMUNE CHECKPOINT BLOCKADE

Most immunotherapeutic strategies, including immune checkpoint inhibition, have shown no or only very limited efficacy in PDA and other cancer types. Neither blockade of CTLA-4, nor PD-L1 induced objective responses in patients with advanced stage PDA. In line with the relatively low immunogenicity of our pancreatic cancer model, reflecting the modest number of somatic mutations in the corresponding clinical indication, the therapeutic impact of anti-PD-1 Ab as monotherapy was very limited. In contrast, the combination of MEKi/PD-1 suppressed the tumor growth in our PDA tumor model with comparable efficiency as MEKi/CD40 Ab. However, long-term control on treatment could only be mediated by MEKi/CD40 Ab, as most of the tumors of the MEKi monotherapy as well as the MEKi/PD-1 combination relapsed within a few weeks after treatment start. One potential reason is that anti-PD-1 Ab, in contrast to anti-CD40 Ab treatment, lacks the capacity induce T cell infiltration and enhancing pro-inflammatory myeloid cells, such as M1 macrophages, which may be essential for T cell dependent long-term anti-tumor effects (**Fig. 2B**).

## CONCLUSIONS AND OUTLOOK

As clinical trials involving drug combination are evolving rapidly, it will be key to select the right combination partners to maximize anti-tumor responses for different cancer entities. In view of this, we are eager to explore the MEKi/CD40 Ab combination in the clinical setting, preferably in the neo-adjuvant setting for the following reasons. First, non-T cell inflamed, ‘cold' tumor can profit the usage of MEKi/CD40 Ab by elevating T cell numbers in the TME prior to resection. Secondly, MEKi treatment can induce immunogenic tumor cell death and together with the CD40 Ab can modulate the immune cell infiltrate towards an immune permissive state. Thirdly, this setting provides the unique opportunity to perform extensive PD biomarker analysis on the treated tumors before proceeding towards extensive clinical efficacy studies. A major handicap of recent immune oncology trials is that PD biomarkers reflecting the *in vivo* impact of sufficient drug exposure in the tumor are missing, making it very difficult to delineate why the anti-tumor impact as observed in pre-clinical studies is not reproduced in the clinical setting. In particular the whole tumor gene signatures offer a promising PD biomarker strategy that can help to bridge pre-clinical and clinical studies, in that it is not confined to the usually limited set of markers but offers a more holistic, unbiased view on drug action.

